# Mitochondrial genome sequence of the protist *Ancyromonas sigmoides* Kent, 1881 (Ancyromonadida) from the Sugluk Inlet, Hudson Strait, Nunavik, Québec

**DOI:** 10.3389/fmicb.2023.1275665

**Published:** 2023-12-08

**Authors:** Romain Gastineau, Sara Harðardóttir, Caroline Guilmette, Claude Lemieux, Monique Turmel, Christian Otis, Brian Boyle, Roger C. Levesque, Jeff Gauthier, Marianne Potvin, Connie Lovejoy

**Affiliations:** ^1^Institute of Marine and Environmental Sciences, University of Szczecin, Szczecin, Poland; ^2^Geological Survey of Denmark and Greenland, København K, Denmark; ^3^Biology Department, Takuvik International Research Laboratory (IRL 3376), Université Laval – CNRS, Québec City, QC, Canada; ^4^The Marine and Freshwater Research Institute in Iceland, Hafnarfjörður, Iceland; ^5^Institut de Biologie Intégrative et des Systèmes, Université Laval, Québec City, QC, Canada; ^6^Département de biochimie, de microbiologie et de bio-Informatique, Université Laval, Québec City, QC, Canada; ^7^Plateforme d’Analyse génomique, Institut de Biologie Intégrative et des Systèmes, Université Laval, Québec City, QC, Canada

**Keywords:** *Ancyromonas sigmoides*, basal protist, Nunavik, Arctic, mitogenome, phylogeny

## Abstract

**Introduction:**

There is little information on evolutionarily ancient eukaryotes, which are often referred to as basal eukaryotes, in Arctic waters. Despite earlier studies being conducted in the Russian White Sea, only few have been reported.

**Methods:**

Following a shotgun sequence survey of diatom cultures from Sugluk Inlet off the Hudson Strait in Northern Québec, we obtained the complete mitochondrial genome and the operon of nuclear ribosomal RNA genes from a strain that matches that of *Ancyromonas sigmoides* (Kent, 1881).

**Results:**

The sequence of the mitogenome retrieved was 41,889 bp in length and encoded 38 protein-coding genes, 5 non-conserved open-reading frames, and 2 rRNA and 24 tRNA genes. The mitogenome has retained *sdh2* and *sdh3*, two genes of the succinate dehydrogenase complex, which are sometimes found among basal eukaryotes but seemingly missing among the Malawimonadidae, a lineage sister to Ancyromonadida in some phylogenies. The phylogeny inferred from the 18S rRNA gene associated *A. sigmoides* from Sugluk Inlet with several other strains originating from the Arctic. The study also unveiled the presence of a metagenomic sequence ascribed to bacteria in GenBank, but it was clearly a mitochondrial genome with a gene content highly similar to that of *A. sigmoides*, including the non-conserved open-reading frames.

**Discussion:**

After re-annotation, a phylogeny was inferred from mitochondrial protein sequences, and it strongly associated *A. sigmoides* with the misidentified organism, with the two being possibly conspecific or sibling species as they are more similar to one another than to species of the genus *Malawimonas.* Overall our phylogeny showed that the ice associated ancryomonads were clearly distinct from more southerly strains.

## Introduction

*Ancyromonas sigmoides* (Kent, 1881) is a zooflagellate protist whose identification and classification have been controversial for more than a century. According to WORMS, the genus *Ancyromonas* encompasses 13 different species from marine and freshwater environments, with seven of them being taxonomically accepted and six with an uncertain status. *Ancyromonas sigmoides*, along with the introduction of the genus *Ancyromonas*, was initially described by Kent (1880–1882) in his “*Manual of the Infusoria*.” The samples that were observed by Kent consisted in ‘decaying fucus’ described as *Fucus silicosa*, probably referring to *Fucus siliquosus* (Linnaeus 1753), today considered a synonym of the taxonomically accepted name *Halidrys siliquosa* (Linnaeus) Lyngbye 1819. These macroalgal samples were picked in the vicinities of Saint Helier, the capital city of the Channel Island Jersey (49.11°N). Since then, several reports have noted the presence of this species worldwide, but solely based on light microscopy, as listed in Cavalier-Smith et al. (2008).

In 1990, Mylnikov (1990) described a colorless flagellate isolated from the Island of Sredni in the White Sea, Republic of Karelia, Russia. He successfully cultivated this strain and shared it with other researchers, including Thomas Cavalier-Smith, and culture collections. Cavalier-Smith et al. carried out the first molecular barcoding of the strain coupled with ultrastructural analyses using transmission electron microscopy (Cavalier-Smith et al., 2008). Based on these results and their suspicions of previous misidentifications, they introduced a taxonomic order called Planomonadida, belonging to the phylum Apusozoa, and redescribed Mylnikov’s strain as *Planomonas mylnikovi* Cavalier-Smith, a species name that was meant to replace *A. sigmoides*.

This reclassification sparked some debate in the community. Heiss et al. (2010) questioned the validity of the genus *Planomonas*, proposed to reverse the transfer of *Planomonas* spp. to their original genera, and declared *P. mylikovi* as a junior synonym of *A. sigmoides*. This new classification was partly accepted by Glücksman et al. (2013) in an article co-authored by Thomas Cavalier-Smith, in which authors agreed to use the species name *A. sigmoides* instead of *P. mylnikovi* to avoid further confusion, but retained the validity of the genus *Planomonas*. Currently, the accepted name of this taxon is *A. sigmoides*, with most of the subsequent work on this species having been conducted on Mylnikov’s strain, generally labeled as B70 or CCAP 1958/3 (e.g., Heiss et al., 2010; Brown et al., 2018).

Finding the phylogenetic position of *A. sigmoides* and to a larger extent of the Ancyromonadida has been challenging. A recent phylogenomic study based on transcriptomic analyses (Brown et al., 2018) has revealed some new insights. The Maximum-Likelihood phylogeny derived from 351 proteins associated the three cultivated species of Ancyromonadida: *A. sigmoides*, Cavalier-Smith 1997 emend Atkins 2000, *Nutomonas longa* Cavalier-Smith & Glücksman 2013, and *Fabomonas tropica* Glücksman and Cavalier-Smith 2013. The phylogeny tended to associate this cluster with Malawimonadidae O’Kelly and Nerad 1999, represented by *Malawimonas jakobiformis* O’Kelly and Nerad 1999, the informally named *Malawimonas californiana*, and a specimen labeled as Malawimonad sp. 249, later formerly described as *Gefionella okellyi* (Heiss et al., 2018). However, the Bayesian inference of phylogeny was not decisive, and Brown et al. (2018) remained cautious with their conclusions, emphasizing the importance of including ancyromonads in further phylogenetic analysis. Missing from these analyses is the genomic information on the mitochondrial genomes of this and other Ancromonadida strains. As mitochondria are a defining feature of eukaryotic cells, knowledge of their phylogeny and genetic architecture is evolutionarily informative and provides basic information on ecology in submarginal habitats (Roger et al., 2017).

In 2018, a strain of diatom identified from light microscopy as *Haslea crucigeroides* (Hustedt) Simonsen, 1974 was isolated from the Sugluk Inlet near the hamlet of Salluit, Nunavik, Québec, Canada. Sagluk Inlet is located along the Hudson Strait, which connects Hudson Bay to Baffin Bay. Genomic sequencing conducted on DNA extracts of the culture revealed the presence of other protists in the culture. A large sequence matching the 18S rRNA gene of *A. sigmoides* was found along with another large contig suspected to represent the mitochondrial genome. Since no reference was available for mitochondrial genes of *A. Sigmoides*, the assignment of this mitogenome to this species was confirmed using previously published transcriptomic data (Brown et al., 2018). Following the annotation of this mitogenome and a general search for similar sequence in GenBank, a sequence derived from the metagenomic analysis that followed the Tara Ocean expedition carried out in open oceans and in the Mediterranean Sea (Tully et al., 2018) was a close match. The sequence had been automatically classified as an “unknown bacteria” in the genus *Rhodovulum* and annotated as such. However, the similarity with the mitogenome of *A. sigmoides* suggested otherwise, and we speculated that it could belong to another species of Ancyromonadida. This sequence was re-annotated and found to have a very similar gene content and phylogenetic proximity with *A. sigmoides*. To the best of our knowledge, the finding of the sequence represents the first confirmed record using molecular techniques of a putative Ancryomonadida in the Mediterranean Sea.

## Materials and methods

### Sampling and cultivation

A diatom identified as *H. crucigeroides* was sampled and isolated from the Sugluk Inlet (62°16′27.66” N, 75°32′3.10” W) in March 2018. The diatom was isolated by micropipette and cultivated in F/2 medium at the Takuvik Laboratory of Université Laval in a Caron culture cabinet model 7901-33-2 (Marietta, OH, United States). The culture cabinet had been modified for cultivating ice algae using low light with continuous irradiance of 9 μmol photons m^−2^ s^−1^ maintained at 0°C. Subsequently, heterotrophic protists were found to be associated with the culture, and they were identified following shotgun sequencing of DNA from the culture. The most prominent co-cultured organism was identified as *A. sigmoides*.

### DNA sequencing and assembly of the genomic data

DNA was extracted with the CTAB protocol following Doyle and Doyle (1990). The library was prepared at the Plateforme d’Analyse génomique, Institut de Biologie Intégrative et des Systèmes, Université Laval (Québec, Canada)^1^ by shearing 500 ng of DNA with a Covaris M220 (Covaris, Woburn MA, United States) and using the dedicated NEBNext Ultra II DNA Library Prep Kit Illumina from New England Biolab (Ipswich, MA, United States). The library was sent to Génome Québec (Montreal QC)^2^ for sequencing on an Illumina (San Diego, CA, United States) NovaSeq 6,000 platform. After sequencing, reads were trimmed using fastp (Chen et al., 2018; Chen, 2023) with the default parameters. After quality trimming, a total of nearly 221 M clean reads remained. Reads were assembled using SPAdes 3.15.4 with the meta mode on and a k-mer of 85, which was found to be optimal in the multispecies culture and provided a good coverage of the *A. sigmoides* mitogenome. The contigs corresponding to the nuclear rRNA cluster and mitochondrial genome of *A. sigmoides* were identified by datamining using standalone blastn and blastx analyses (Boratyn et al., 2012) with customized databases containing nucleotide and protein sequences from diatoms, prepared using the make blastdb function of the NCBI package. All bioinformatic analyses were conducted on the superdome flex server at IBIS, Université Laval.

### Transcriptomic assembly

The RNA-Seq data from Brown et al. (2018) on *A. sigmoides* B70/CCAP 1958/3 (SRR5997436) were downloaded from the NCBI Sequence Read Archive (SRA). After conversion to fastq format by SRA Toolkit,^3^ reads were assembled using RNASPAdes 3.15.4 (Bushmanova et al., 2019) with a k-mer of 35. The resulting contig file was datamined by a command line blastn analysis using the putative *cox1* gene sequence of *A. sigmoides* from Sugluk Inlet.

### Annotation of the nuclear rRNA cluster and mitochondrial genome

The rRNA cluster was analyzed using Rfam (Kalvari et al., 2021). Annotation of the protein coding genes (PCG) of mitochondrial genomes was done with genetic code 11 using the findORF tools developed at Université Laval (Gagnon, 2004) and used to annotate organellar genomes in several recent publications (for, e.g., Hamedi et al., 2019; Gastineau et al., 2021; and Solak et al., 2021). The database on which findORF operates contains sequences from the gene-rich mitochondrial genome of the early emerging (basal) protists *Jakoba libera* (Ruinen) D.J. Patterson 1990 ATCC 50422 (GenBank: KC353355), *Reclinomonas americana* Flavin & Nerad, 1993 (GenBank: KC353356), and *Histiona aroides* Pascher 1943 (GenBank: KC353353), from Leigh and Lang (2004), Seif et al. (2006), and Burger et al. (2013). The positions of tRNA genes were found using ARWEN (Laslett and Canbäck, 2008). Finally, the maps of the mitochondrial genomes were drawn using the OrganellarGrenomeDRAW (OGDRAW v. 1.3.1) portal (Greiner et al., 2019).

### Phylogenies

For the 18S inferred phylogeny, the dataset from Glücksman et al. (2013) was appended with the 18S gene of *A. sigmoide*s from Sugluk Inlet. Sequences were aligned using MAFFT 7 (Katoh and Standley, 2013; auto option) and trimmed using trimAl (Capella-Gutiérrez et al., 2009; automated1 option); the resulting alignment was 1,613 bp long. The best model of evolution (TrN + I + G4 model) was obtained on this alignment using Model-Test-NG (Darriba et al., 2020) with the default parameters. This model works well for datasets originating from different types of genomes and is used to find the best model on separate alignments before concatenation. The Maximum-Likelihood phylogenetic analysis was conducted using IQ-TREE v.2.2.0 (Minh et al., 2020) with 10,000 ultra-fast bootstrap replicates. Following the protocol in Glücksman et al. (2013), the tree was rooted with taxa representing Mantamonadida and Apusomonadida.

For the mitochondrial protein inferred phylogeny, amino-acid sequences of the proteins encoded by *atp6*, *atp8*, *atp9*, *cob*, *cox2*, *nad1*, *nad2*, *nad3*, *nad4*, *nad4L*, *nad5*, *nad6*, and *nad9* were extracted from the mitogenomes of *A. sigmoides*, *M. jakobiformis* (AF295546), *M. californiana* (KP165387), *C*. *okellyi* (represented by two contigs, KP165390 + KP165391), and *J. libera* (KC353355) as well as from the sequence registered as PALT01000012 on GenBank after it had been curated based on our findORF analysis, as explained in the Results section. The protein sequences used for the phylogeny were selected according to three criteria: they are present in all species (excluding, for example, genes of the complex II), not fused into a single open-reading frame (thus excluding *cox1* and *cox3*), and easily alignable (excluding, for example, *atp4*). The protein sequences were aligned separately using MAFFT 7, trimmed using trimAl (identical options), and then concatenated using Phyutility 2.7.1 (Smith and Dunn, 2008). The size of the resulting alignment was 3,482 amino acids. The best model of evolution, the MTZOA+G4 + F model, was obtained with Model-Test-NG using the default parameters on the concatenated alignment. The phylogeny was inferred using IQ-TREE v.2.2.0 with 1,000 ultra-fast bootstrap replicates.

### Datamining the TARA metagenomic data from Arctic seawater

We then searched the nine files containing the metagenomic co-assemblies obtained in the frame of the TARA project from the Arctic Ocean and sub-Arctic North Atlantic Ocean samples (project PRJEB41575) downloaded from ENA.^4^ Each file was submitted to a blastn query using the mitogenome of *A. sigmoides* with a E-value filter of 1e^−100^. Contigs of interest were extracted from the assembly files and submitted to megablast queries online to test for possible extended northern distribution of the species.

## Results

### Nuclear rRNA operon

After assembly and trimming, a 5,062 bp contig was obtained and its sequence was deposited on GenBank (OR380967). The sizes of the different components of the operon as identified by Rfam are listed in Table 1. It is unclear whether the 28S gene was complete, but we failed at extending the fragment further.

**Table 1:** Sizes of the different genes and internal transcribed spacers in the rRNA operon of *Ancyromona sigmoides* from the Sugluk Inlet.

Part of the rRNA operon	18S	ITS1	5.8S	ITS2	28S
Size in bp	1,799	246	151	381	2,485

The 28S gene might not be complete.

The BLASTn analysis of the 18S gene showed 98.66% similarity (2 polymorphisms) with the sequence EU349231 obtained by Cavalier-Smith et al. (2008) from the B70/CCAP 1958/3 reference strain. The whole cluster was 98.66% similar to the sequence MW872731, which corresponds to the partial 3,363 bp rRNA operon cluster from the same strain as the EU349231 sequence. Most of the differences were within the internal transcribed spacers 1 (92.21% similarity) and 2 (92.61% similarity) and consisted of both SNPs and indels.

### The 18S-inferred phylogeny

**Figure 1:**
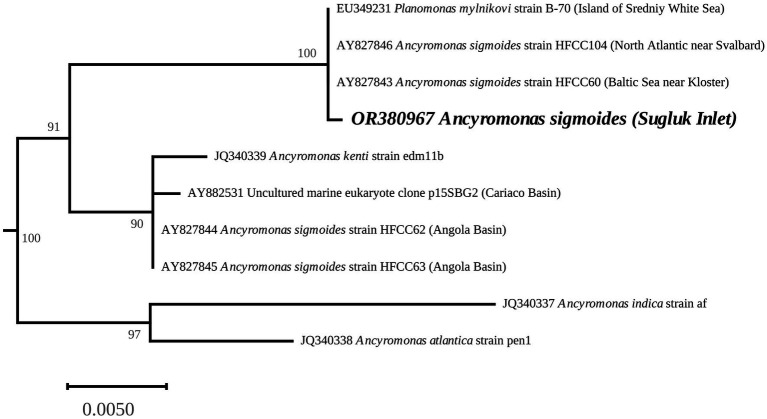
*Ancyromonas* spp. phylogenetic position, derived from the 18S rRNA gene-inferred Maximum-Likelihood tree. The values at the nodes indicate the support. The scale represents the number of substitutions per site. The complete phylogeny was performed with 34 taxa (doi: 10.5281/zenodo.8208290). The complete tree was rooted with the cluster containing Apusomonadida and Mantomonadida.

A detailed tree of available Apusomonadida 18S rRNA sequences (available as a Newick file; see data availability statement) distinguished a highly supported branch (cluster) containing *Ancyromonas* spp. (Figure 1). It clearly associates *A. sigmoides* from Sugluk Inlet with other strains, including the type material from Cavalier-Smith et al. (2008). As previously observed by Glücksman et al. (2013), specimens from the Northern Hemisphere, including the Baltic Sea, the vicinities of Svalbard, Sugluk Inlet, and the White Sea can be distinguished from other strains that were described as *A. sigmoides* from the Angola Basin in Scheckenbach et al. (2005) and Wylezich et al. (2010). The latter strains appear closer to *Ancyromonas kenti* Glücksman and Cavalier-Smith as defined by Glücksman et al. (2013). It should be noted that the names (nomenclature) in the tree used for the species correspond to the original GenBank references and were not modified, as mentioned by Glücksman et al. (2013).

### The mitochondrial genome

As the small multiple copies of mitogenomes can be retained in RNAseq data, we performed a BLASTn analysis using the putative *cox1* gene sequence of *A. sigmoides* as a query against the assembled transcriptome from Brown et al. (2018) returned a 5,466 bp sequence with a coverage of 345.26X. This contig derived from the assembled transcriptome perfectly aligned with our assembled mitogenome. For the corresponding *cox1* gene, a total of 12 SNPs out of 1986 bp were found, leading to a single amino-acid change in the predicted protein sequence.

**Figure 2:**
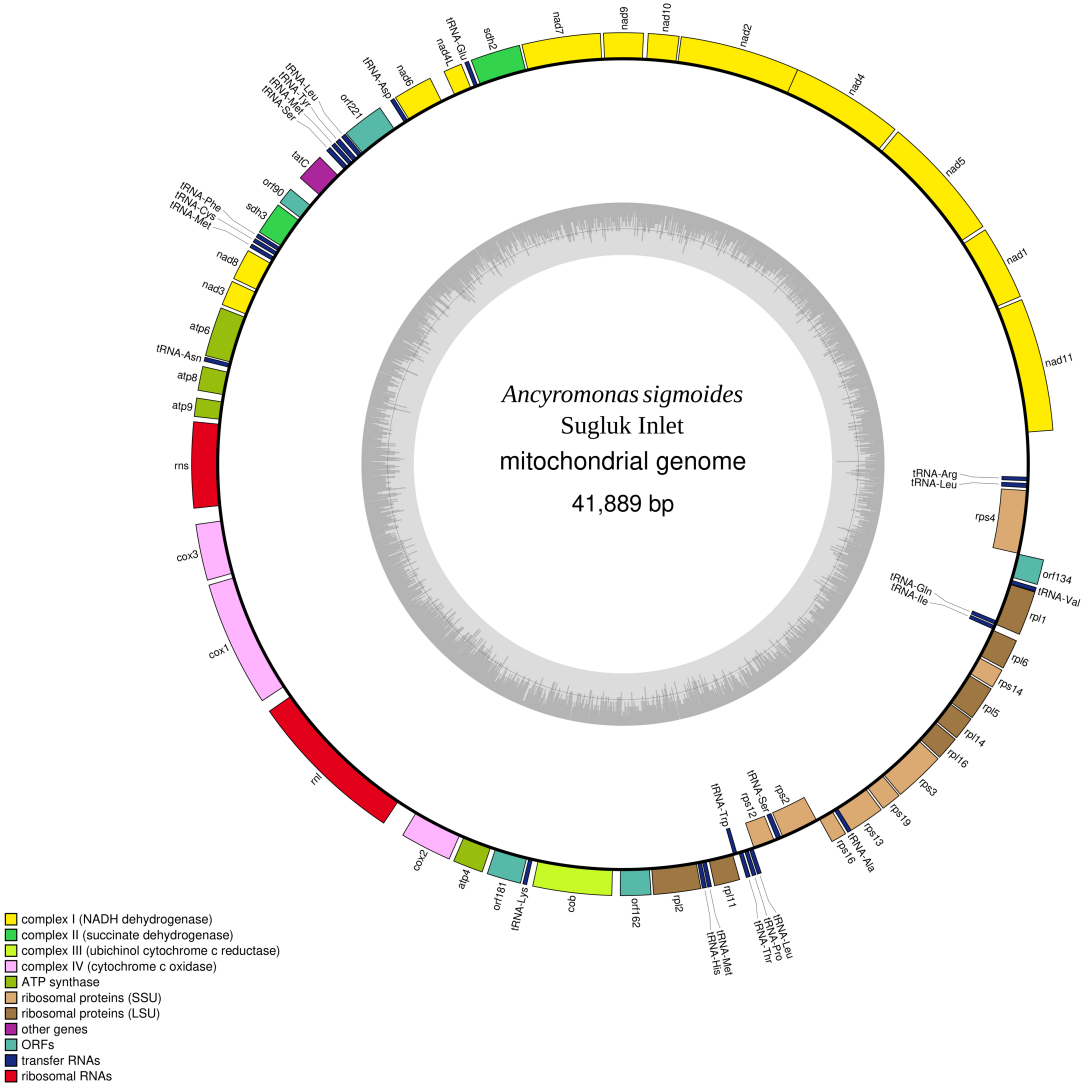
Map of the mitochondrial genome of *Ancyromonas sigmoides* from the Sugluk Inlet.

The contig identified as the mitogenome of *A. sigmoides* is 41,889 bp long (GenBank: OR393254). Because of the suspected presence of repetitions at its ends, the complete mitogenome may be larger; however, to facilitate reading, we have represented the mitogenome as circular (Figure 2). Annotation of the protein coding genes (PCG) was done using code 11, with all PCG starting with ATG except for *rpl1* (starts with GTG). The genome is gene-rich and encodes 38 PCG and five non-conserved open-reading frames (ORF). Two rRNA genes were detected (*rns* and *rnl*) but we failed to identify a putative 5S rRNA gene. A total of 24 tRNA genes were also found. The detailed gene composition is provided in Table 2 and Figure 2.

**Table 2:** Gene composition of the mitochondrial genome of *Ancyromona sigmoides* from Sugluk Inlet.

Type of gene	Gene content
complex I (NADH dehydrogenase)	*nad1*, *nad2*, *nad3*, *nad4*, *nad4L*, *nad5*, *nad6*, *nad7*, *nad8*, *nad9*, *nad10*, *nad11*
complex II (succinate dehydrogenase)	*sdh2*, *sdh3*
complex III (ubichinol cytochrome c reductase)	*cob*
complex IV (cytochrome c oxidase)	*cox1*, *cox2*, *cox3*
ATP synthase	*atp4*, *atp6*, *atp8*, *atp9*
ribosomal proteins (SSU)	*rps2*, *rps3*, *rps4*, *rps12*, *rps13*, *rps14*, *rps16*, *rps19*
ribosomal proteins (LSU)	*rpl1*, *rpl2*, *rpl5*, *rpl6*, *rpl11*, *rpl14*, *rpl16*
other genes	*tatC*
ORFs	*orf90*, *orf134*, *orf162*, *orf181*, *orf221*
transfer RNAs	*tRNA-Glu(ttc), tRNA-Asp(gtc), tRNA-Leu(tag), tRNA-Tyr(gta), tRNA-Met(cat), tRNA-Ser(tga), tRNA-Phe(gaa), tRNA-Cys(gca), tRNA-Met(cat), tRNA-Asn(gtt), tRNA-Lys(ttt), tRNA-His(gtg), tRNA-Met(cat), tRNA-Trp(cca), tRNA-Thr(tgt), tRNA-Pro(tgg), tRNA-Leu(caa), tRNA-Ser(gct), tRNA-Ala(tgc), tRNA-Ile(gat), tRNA-Gln(ttg), tRNA-Val(tac), tRNA-Leu(taa), tRNA-Arg(tct)*
ribosomal RNAs	*rnL*, *rnS*

Among the 24 tRNA gene products, 19 had a normal cloverleaf structure and 5 had a long variable loop (2 tRNA-Leu, 2 tRNA-Ser, and 1 tRNA-Tyr). It is worth noting that three tRNA-Leu (different anticodons), three tRNA-Met (identical anticodons), and two tRNA-Ser (different anticodons) were detected.

### Comparison with Malawimonadidae

No inverted repeat-like structure was detected in *A. sigmoides*, unlike the mitogenome of *M. jakobiformis*.^5^ The *A*. *sigmoides* mitogenome was richer than any of its homologs in the Malawimonadidae, in terms of NADH dehydrogenase subunit genes, as it also contained *nad7*, *nad8*, *nad10*, and *nad11*. The number of ribosomal protein genes appears to be variable, with the lowest number being recorded for *G. okellyi*. *A. sigmoides* appears to be the only species to encode *rps16* and *rpl1*. A *tatC* gene, coding for a putative SecY-independent transporter protein, was found only in *A. sigmoides* and *M. jakobiformis*. Both species of *Malawimonas* spp. contain *ccmF* and *ccmC* genes. All four species (*A. sigmoides*, *M. jakobiformis*, *M. californiana* and *G. okellyi*) display the three cytochrome c oxidase genes, *cox1*, *cox2*, and *cox3*; however, *G. okellyi* is the only one in which *cox1* and *cox3* are fused into a single ORF. Finally, *A. sigmoides* is the only species in the phylogeny whose mitogenome has retained two genes related to the succinate dehydrogenase complex, namely, *sdh2* and *sdh3*.

### Comparison with the Tara Ocean metagenome sequence PALT01000012

**Figure 3:**
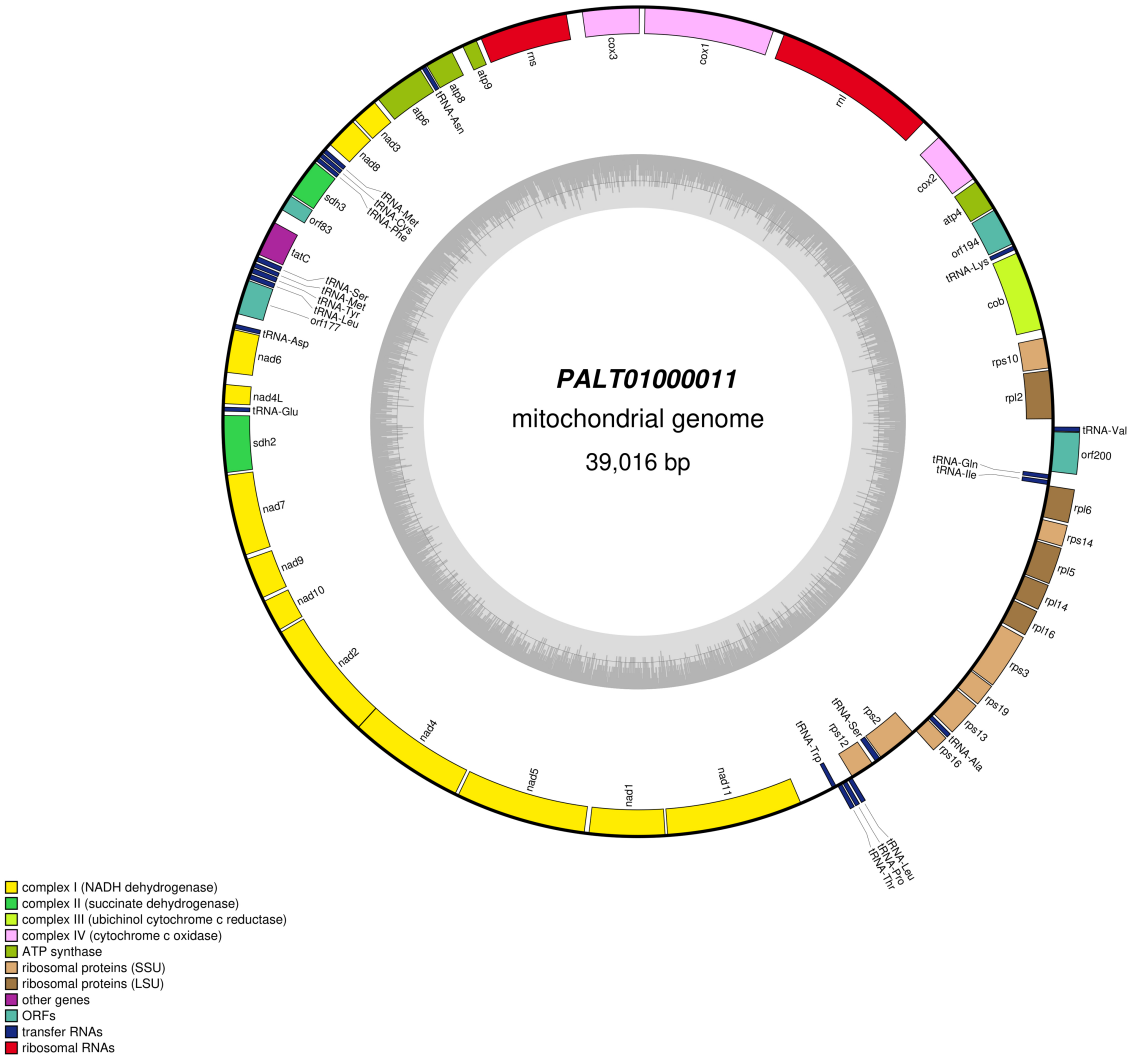
Map of the mitochondrial genome of the sequence labeled in GenBank as PALT01000011 after re-annotation.

Our blastp queries of the Sdh2 and Sdh3 proteins from *A*. *sigmoides* returned as the best-matched proteins ascribed to *Rhodovulum* sp., with e-values/identities of 7^e-147^/78.12% and 9^e-23^/35.26%, respectively. A deeper enquiry on the genome sequence from which these proteins were derived showed that it corresponds to a 39,016 bp contig of a metagenomic assembly (PALT01000012) based on the Mediterranean Sea samples collected in the frame of the Tara Ocean (Tully et al., 2018). The TARA data were automatically annotated using the NCBI Prokaryotic Genome Annotation Pipeline. The contig’s size, gene content, and gene organization strongly suggested that it might instead belong to the mitochondrial genome of a basal eukaryote, likely an Ancyromonadida. We annotated this sequence using the same tools and parameters as for *A. sigmoides*. The gene content appeared nearly identical when compared to the *A. sigmoides* mitogenome (Figure 3). All genes of the complexes I to IV were shared by the two mitogenomes. The differences were observed for the ribosomal proteins, as *rps10* was not found in *A. sigmoides* by the annotation tool but possibly corresponding to orf162. In contrast, *rps4* and *rpl1* were not found in PALT01000012 but could possibly correspond to orf200 in the case of *rpl1*. In addition, we noted that some unidentified ORFs clustered with conserved protein-coding genes within both genomes. This is the case for orf221/orf177, orf181/orf194, and orf90/orf83 in *A. sigmoides*/PALT01000012. Although poorly informative, this might still be an indication that these ORFs correspond to functional genes that remain to be identified (Table 3).

**Table 3:** Comparison between the protein-coding genes identified and open-reading frames found in the mitochondrial genomes of *Ancyromonas sigmoides*, *Malawimonas jakobiformis*, *Malawimonas californiana*, and *Gefionella okellyi*.

Species	*Ancyromonas sigmoides*	*Malawimonas jakobiformis*	*Malawimonas californiana*	*Gefionella okellyi*
GenBank Accession No	OR393254	AF295546	KP165387	KP165390 + KP165391
Size of the mitogenome	41,889	47,328 bp	36,792	20,798 bp + 19,770 bp
Genes identified in the mitochondrial genome				
complex I	*nad1, nad2, nad3, nad4, nad4L, nad5, nad6,**nad7**,**nad8**, nad9,**nad10**,**nad11***	*nad1, nad2, nad3, nad4L, nad4, nad5, nad6, nad9,*	*nad1, nad2, nad3, nad4L, nad4, nad5, nad6, nad9*	*nad1, nad2, nad3, nad4L, nad4, nad5, nad6, nad9*
complex II	***sdh2**,**sdh3***	None	None	
complex III	*cob*	*cob*	*cob*	
complex IV	*cox1, cox2, cox3*	*cox1, cox2, cox3*	*cox1, cox2, cox3*	*cox1/cox3* fused, *cox2*
ATP synthase	*atp4, atp6, atp8, atp9*	atp4 (ymf39)*, atp6, atp8, atp9	*atp4, atp6, atp8, atp9*	*atp4, atp6, atp8, atp9*
ribosomal proteins (SSU)	*rps2, rps3, rps4, rps12, rps13, rps14,**rps16**, rps19*	*rps1, rps2, rps3, rps4, rps7, rps8, rps11, rps12, rps13, rps14, rps19,*	*rps2, rps3, rps4, rps7, rps8, rps11, rps12, rps13, rps14, rps19,*	*rps3, rps4, rps7, rps11, rps12, rps13, rps19*
ribosomal proteins (LSU)	***rpl1**, rpl2, rpl5, rpl6, rpl11, rpl14, rpl16*	*rpl2, rpl5, rpl6, rpl11, rpl14, rpl16, rpl18, rpl19, rpl20, rpl31, rpl36*	*rpl2, rpl5, rpl6, rpl11, rpl14, rpl16, rpl19, rpl20*	*rpl5, rpl11, rpl14, rpl19*
other genes	*tatC*	*Ccmf (yejR), ccmC (yejU), yejV, tatC (ymf16)*	*ccmC, ccmF*	
Non-conserved open-reading frames found in the mitochondrial genome				
ORFs	orf90, orf134, orf162, orf181, orf221	orf120, orf332, orf74,	orf129, orf209	Orf131, orf192, orf285, orf335, orf488

The genes in bold have been found only in *A. sigmoides*. *refers to Burger et al. (2003).

### Mitochondrial protein inferred phylogeny

**Figure 4:**
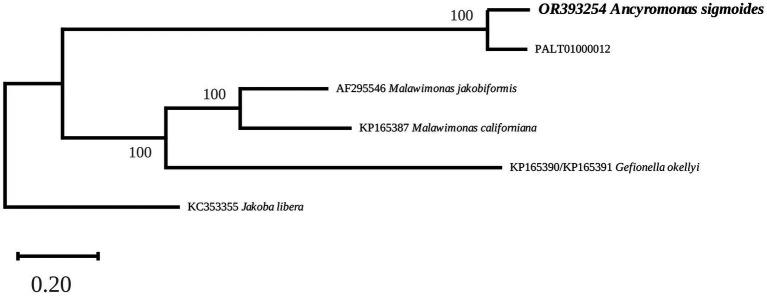
Maximum-Likelihood phylogenetic tree inferred from an alignment of 13 protein sequences. The values at the nodes indicate the support. The scale represents the number of substitutions per site. *Jakoba libera* was used as an outgroup.

The phylogenetic tree clearly associated *A. sigmoides* with PALT01000012, separating them from Malawimonadidae, which formed a highly supported cluster of their own (Figure 4). However, the tree did not display bootstrap values for the node separating Ancyromonadida and Malawimonadidae. To check whether this result could be linked to the software used, a separate phylogenetic analysis of the same dataset was carried out using RAxML 8.0 (Stamatakis, 2014) with the MTZOA+G4 model of evolution. The best tree computed for 100 bootstrap replicates led to identical results. It is thus likely that, at this taxonomic scale, our dataset was not sufficient to resolve phylogenetic relationships, as pointed out by Brown et al. (2018). Nevertheless, the proximity between *A. sigmoides* and PALT01000012 supports our assumption that the latter sequence belongs to the mitochondrial genome of Ancyromonadida.

### Searching for other Ancryomonadida in the Arctic Tara data

We attempted to use the filtering parameters described above, to assess the presence of *A. sigmoides* among the metagenomes in PRJEB41575 from the Arctic Tara data. Most of the contigs returning positive mitochondria results belonged to various stramenopiles, including diatoms while others showed limited identity (approximately 85%) with an unidentified organism of the order Telonemida Cavalier-Smith 1993 (MN082145). A single fragment could possibly be ascribed to a protist related to Ancyromonadida. This fragment from the biosample SAMEA11025526, analysis ERZ4109989, was from co-assembly of multiple station mesopelagic and mixed layer samples. The contig labeled as k117_6354597 was 2,156 bp long and returned a best megablast query to *A. sigmoides* with e value 4e^−110^ and 85.43% identity. The contig contained three ORFs that correspond to *nad9*, *nad10*, and *atp8*. Blastp queries of these ORFs returned as best results *A. sigmoides* followed by P ALT01000012 as discussed above. It is worth noting that, although *nad9* and *nad10* are adjacent in *A. sigmoides* and PALT01000012, *atp8* is not. Overall, the organism represented by contig k117_6354597 was distant from the two others.

## Discussion

To the best of our knowledge, it was possible to obtain, characterize, and annotate the complete mitochondrial genome of a sub-arctic-Arctic *A. sigmoides* for Ancyromonadida. In the near future, we hope to isolate *A. sigmoides* from the diatom culture and cultivate it as a monoclonal strain for further investigation.

Unlike the mitogenomes of Malawimonadidae, the mitogenomes of *A. sigmoides* has retained two succinate dehydrogenase genes (*sdh2* and *sdh3*). These genes have been found in the mitogenomes of other ancient lineages, such as the Jakobida, which include the previously mentioned *J. libera*, *H. aroides*, and *R. americana*. In these species, it is worth noting that three *sdh* genes are present, the third being *sdh4*. It is possible that one of the non-identified ORFs of *A. sigmoides* represents a poorly conserved version of this gene; however, in the absence of deeper investigation, it can also be speculated that this gene has been lost or more likely transferred to the nuclear genome. Sequencing additional mitochondrial genomes of Ancyromonadida should provide clues regarding the presence and number of succinate dehydrogenase genes among them.

This is not the first time that specimens ascribed to *A*. *sigmoides* have been found in cold waters of the North, as illustrated in our 18S-inferred phylogeny. Beyond the strain isolated by Mylnikov, a specimen labeled as *A. sigmoides* strain HFCC104 by Scheckenbach et al. (2005) and Wylezich et al. (2010) was isolated off the western coast of Svalbard. Based on our findings and the research conducted by Glücksman et al. (2013) it is evident that the Arctic and Northern Hemisphere strains are distinctly different from those found in the Angola Basin, which were labeled as *A*. *sigmoides* by Scheckenbach et al. (2005, 2006) but mentioned as *Ancyromonas* sp. by Glücksman et al. (2013). Similarly, the previous studies conducted by Scheckenbach et al. (2006) led to separate freshwater and seawater strains ascribed to *A. sigmoides* based on molecular phylogeny. It is possible that further studies will reveal the cryptic diversity among Ancyromonadida, with *A. sigmoides* having geographically restricted representatives in the Arctic and North Atlantic regions, as previously observed in algal isolates (Dorrell et al., 2022). The association with a sea-ice diatom culture is also intriguing and could suggest that this strain is potentially a mutualistic partner or first year ice specialist, and as such, of relevance for this threatened Arctic ecosystem. The use of a mitochondrial genome has advantages, as mitochondrial genes evolve faster than nuclear rRNA genes, thus enabling work at the population level of a species. With this aim, it would be informative to compare the mitogenomes of different strains of *A*. *sigmoides* from the Arctic and North Atlantic regions.

Finally, our study suggests that there might be unsuspected genomic information in GenBank that cannot be reached by taxonomical lineage queries on the database. When such results from general blastn searches are anomalous, a second deeper exploration of these sequences is worth carrying out, and the annotation could be verified with the tools dedicated to eukaryotes. In our case, we showed the conserved presence of the two succinate dehydrogenase genes and non-identified ORFs clustering with conserved PCG within the sequences of our Sugluk Inlet *A. sigmoides* and an environmental construct. Future studies could extend targeted datamining; searching for additional mitogenomes belonging to diverse protists in the TARA Arctic metagenomes and other large environmental databases.

## Data availability statement

The datasets presented in this study can be found in online repositories. The names of the repository/repositories and accession number(s) can be found at: https://www.ncbi.nlm.nih.gov/genbank/, OR380967 and OR393254.

## Author contributions

RG: Investigation, Writing – original draft. SH: Investigation, Writing – review & editing. CG: Investigation, Writing – review & editing. CLe: Investigation, Writing – review & editing. MT: Investigation, Writing – review & editing. CO: Investigation, Writing – review & editing. BB: Investigation, Writing – review & editing. RL: Investigation, Writing – review & editing. JG: Investigation, Writing – review & editing. MP: Investigation, Writing – review & editing. CLo: Funding acquisition, Project administration, Supervision, Writing – original draft, Writing – review & editing.

## Footnotes

^1^ https://www.ibis.ulaval.ca/en/services-2/genomic-analysis-platform/

^2^ https://www.genomequebec.com/

^3^ https://github.com/ncbi/sra-tools

^4^ https://www.ebi.ac.uk/ena/browser/view/PRJEB41575

^5^ https://megasun.bch.umontreal.ca/ogmp/projects/mjako/gen.html
